# Isolation and sequence analysis of the wheat B genome subtelomeric DNA

**DOI:** 10.1186/1471-2164-10-414

**Published:** 2009-09-05

**Authors:** Elena A Salina, Ekaterina M Sergeeva, Irina G Adonina, Andrey B Shcherban, Dmitry A Afonnikov, Harry Belcram, Cecile Huneau, Boulos Chalhoub

**Affiliations:** 1 Institute of Cytology and Genetics, Siberian Branch of the Russian Academy of Science, pr. Lavrentieva 10, Novosibirsk, 630090 Russia; 2 UMR INRA 1165 - CNRS 8114 UEVE - Unite de Recherche en Genomique Vegetale (URGV), 2, rue Gaston Cremieux, CP5708, 91057 Evry cedex, France

## Abstract

**Background:**

Telomeric and subtelomeric regions are essential for genome stability and regular chromosome replication. In this work, we have characterized the wheat BAC (bacterial artificial chromosome) clones containing Spelt1 and Spelt52 sequences, which belong to the subtelomeric repeats of the B/G genomes of wheats and *Aegilops *species from the section *Sitopsis*.

**Results:**

The BAC library from *Triticum aestivum *cv. Renan was screened using Spelt1 and Spelt52 as probes. Nine positive clones were isolated; of them, clone 2050O8 was localized mainly to the distal parts of wheat chromosomes by *in situ *hybridization. The distribution of the other clones indicated the presence of different types of repetitive sequences in BACs. Use of different approaches allowed us to prove that seven of the nine isolated clones belonged to the subtelomeric chromosomal regions. Clone 2050O8 was sequenced and its sequence of 119 737 bp was annotated. It is composed of 33% transposable elements (TEs), 8.2% Spelt52 (namely, the subfamily Spelt52.2) and five non-TE-related genes. DNA transposons are predominant, making up 24.6% of the entire BAC clone, whereas retroelements account for 8.4% of the clone length. The full-length CACTA transposon *Caspar *covers 11 666 bp, encoding a transposase and CTG-2 proteins, and this transposon accounts for 40% of the DNA transposons. The *in situ *hybridization data for 2050O8 derived subclones in combination with the BLAST search against wheat mapped ESTs (expressed sequence tags) suggest that clone 2050O8 is located in the terminal bin 4BL-10 (0.95-1.0). Additionally, four of the predicted 2050O8 genes showed significant homology to four putative orthologous rice genes in the distal part of rice chromosome 3S and confirm the synteny to wheat 4BL.

**Conclusion:**

Satellite DNA sequences from the subtelomeric regions of diploid wheat progenitor can be used for selecting the BAC clones from the corresponding regions of hexaploid wheat chromosomes. It has been demonstrated for the first time that Spelt52 sequences were involved in the evolution of terminal regions of common wheat chromosomes. Our research provides new insights into the microcollinearity in the terminal regions of wheat chromosomes 4BL and rice chromosome 3S.

## Background

Two regions are distinguished in the chromosomal end structure: the telomeric region that caps the chromosome tip and the adjacent subtelomeric region. Taken together, the results of recent analyses of subtelomeric DNA and the current views suggest that the subtelomeric region is located in the distal chromosomal region between the telomeric and unique chromosome-specific DNA sequences [1-3]. On the other hand, it should be taken into account that not all species and not all chromosomes have unique chromosome-specific DNA sequences in their distal regions; this is especially true for the polyploid species. There is a vast literature concerning the structure and function of telomeric DNA as a specialized end structure in a wide range of eukaryotes. Briefly, this DNA consists of the (TTAGGG)_n_-like sequences and is associated with specific nucleosomal proteins, which provide the telomere protection function and regulation of telomere tract length [4,5]. The first plant telomeric DNA was isolated and cloned from *Arabidopsis thaliana *[6]. The *Arabidopsis*-type telomere TTTAGGG is conserved and widely occurs among plants; however, it has not been found in *Alliaceae *as well as in many other Asparagales [7,8]. The isolation and research of distinct repetitive DNA families located at the chromosomal ends have been so far widely used in analysis of subtelomeric plant DNA. It has been repeatedly demonstrated that the subtelomeric regions of the chromosomes in plant taxa are composed of various tandem repeat families, some of which are species- and/or genome-specific [9-12]. In *Secale cereale *and *Aegilops speltoides*, the species-specific subtelomeric families of tandem repeats constitute about 2% of the nuclear DNA [13,14]. The measured lengths of various subtelomeric tandem repeats, their variation patterns, and proximity to telomeric repeats have been analyzed in detail in tomato, barley, rye, and rice. In particular, it has been shown that subtelomeric and telomeric repeats are co-localized on DNA fragments longer than 300 kb in rice [10]. Based on the distance between two FISH (fluorescent *in situ *hybridization) signals, the distance between subtelomeric and telomeric repeats on some rice chromosomes was estimated as less than 100 kb [15]. The experiments with stretched rye chromosome fibrils have clearly demonstrated that in certain case, the distance between a long telomeric repeat and the immediately adjacent copies of the subtelomeric repeat pSc250 was less than 4 kb [16]. Variations in the distance between telomeric repeats and the subtelomeric satellites following them have been also observed in tomato [17,18]. It is of interest that the studies of subtelomeric regions in rye and *Aegilops *have detected diverse combinations of tandemly arranged subtelomeric repeats with interspersed non-repetitive sequences [[16], Salina et al. (unpublished data)]. It has been shown that mutual arrangement of subtelomeric repeats and the presence of interspersed unique or low copy number sequences are chromosome-specific, providing *Silene latifolia *as an example [19]. Use of a degenerate telomere primer and the Vectorette PCR approach has made it possible to isolate and map the DNA sequences adjacent to telomeric repeats. Cloning of telomere associated sequences in barley has demonstrated that in some cases telomeric repeats are immediately adjacent to subtelomeric tandem repeats [20]. Large-scale sequencing of the genomes of a number of organisms enabled to completely characterize the structural organization of the subtelomeric regions in almost all human chromosomes [21,22] and certain rice [23] and *Arabidopsis *chromosomes [3]. The salient findings include multiple segmental duplications occurring in more than one subtelomeric region, the presence of mobile elements, chromosome-specific tandem repeats, abundance of internal (TTAGGG)_n_-like sequences, the presence of transcribed regions, and the putative genes in subtelomeric regions.

The BAC libraries have been recently created and used intensively for studying the genome of *Triticum aestivum *[24]. This has offered unprecedented opportunities for examining, in particular, the extensive subtelomeric DNA regions of three homoeologous genomes (BB, AA, and DD) to get the insights into their reshuffling during formation of the allopolyploid nucleus and evolution. Two approaches can be used for selecting the subtelomeric BAC clones, namely, (1) with the help of telomere-specific probes, providing for choosing the clones that contain telomeric repeats and the associated sequences, and (2) using as probes the DNA sequences localized by *in situ *hybridization to the chromosome ends.

Spelt1 and Spelt52 are the satellite DNA sequences detected by *in situ *hybridization on the chromosome ends of several diploid and polyploid *Triticum *and *Aegilops *species. Spelt1 sequences are the genome-specific subtelomeric tandem repeats of *Ae. speltoides *(2*n *= 14), the putative progenitor of the B and G genomes in polyploid wheats [12]. The number of Spelt1 localization sites on the chromosome ends of this species is mainly 24-28 per genome, although some accessions contain smaller number of these sites. The copy number of Spelt1 is considerably decreased in the genomes of polyploid species; the maximal number of hybridization sites, amounting to 12, is detected on the chromosome ends of the tetraploid wheat *T. timopheevii *(GGA^t^A^t ^genome). In the tetraploid (BBAA) and hexaploid (BBAADD) wheats of the Emmer group, the number of the loci containing these repeats varies from zero to six and Spelt1 is localized to the ends of predominantly B genome chromosomes [12].

Spelt52 is localized to the subtelomeric chromosome regions of the three of five diploid species from the section *Sitopsis *(*Ae. speltoides*, *Ae. longissima*, and *Ae. sharonensis*) and is undetectable in the genomes of the remaining diploid species, progenitors of the A and D genomes of hexaploid wheat. In *Ae. speltoides*, Spelt52 is in the subtelomeric regions of the majority of chromosomes; however, the number of chromosomes containing this repeat decreases to three in the tetraploid wheats of the *Timopheevi *group. Neither Spelt52 nor homologous sequences were detectable by *in situ *hybridization in the Emmer wheats, including soft wheat [12,25].

Both subtelomeric sequences, Spelt1 and Spelt52, are always detectable by PCR and Southern hybridization in the genome of wheat even when *in situ *hybridization fails to identify these repeats on the chromosomes [26].

The aim of this research was to analyze the organization of subtelomeric genomic regions containing the Spelt1 and Spelt52 sequences in the B genome of hexaploid wheat.

## Results

### Identification of the BAC clones with Spelt1 and Spelt52 sequences

The location of Spelt1 and Spelt52 at chromosomal ends and low copy number in the *T. aestivum *genome were the advantages enabling Spelt1 and Spelt52 to be used as probes for selecting appropriate subtelomeric BAC clones. We screened the *T. aestivum *(cv. Renan) BAC genomic libraries, representing 8× genome equivalents, with 1 032 192 clones. The first step consisted in PCR screening of pools of BAC clones. We used the universal Spelt1(F/R) and Spelt52(F/R) primers (see Materials and Methods). The PCR pattern corresponding to a multiband amplification, characteristic of tandemly organized DNA sequences, was obtained for five BAC clones with Spelt52(F/R) primers. These five BAC clones were used for further characterization (Table 1). We failed to identify Spelt1-containing BAC using this PCR screening strategy. Therefore, we hybridized part of the library (290 000 BAC clones) with Spelt1 probe and identified four BAC clones for further analysis (Table 1).

**Table 1:** The results of comparative BAC-clone analysis

**BAC clone no.**	**Selected as containing the following sequence**	**Length, kb**	**Content of repetitive sequences**				Approximate content of repetitive sequences (%)**	***In situ *hybridization data**
					
			Copy number*		Presence**			
					
			**Spelt1** **178 bp**	**Spelt52** **380 bp**	**pSc 119.2**	**pAs1**		
2322J20	Spelt1	93	7	-	-	-	54	***
		
2383A24		113.5	6	-	-	-	80	Dispersed
		
2424P01		97	5	-	-	-	70	Dispersed
		
112D20		86.5	1-2	5	-	-	62	Dispersed

110O07	Spelt52	162.5	-	24	-	-	56	Dispersed
		
110B21		168	-	23	-	-	73	Dispersed
		
2050O8		120	-	27	-	-	25	Subtelomeric
		
478L20		153.5	-	27	-	+	33	***
		
1487N20		165.5	-	28	-	-	62	Dispersed

* Estimated by Southern and dot blot hybridizations, the monomer length is specified for each repeat family; ** estimated by Southern blot hybridization; and *** long fragment isolated from the clone has a subtelomeric localization.

### Characterization of candidate subtelomeric clones

The BAC clones identified as containing Spelt1 and Spelt52 repeats were further characterized as follows:

(1) Determination of insert lengths;

(2) Estimation of the content of repetitive sequence in cloned DNA fragments, including various repeats occurring in the subtelomeric regions in wheat--Spelt1 [14], Spelt52 [26], pSc119.2 [27], pAs1 [28], and the telomeric repeat TTTAGGG [6]; and

(3) Hybridization of BAC clones to mitotic metaphase chromosomes of the common wheat cvs. Chinese Spring (CS) and Renan.

**Figure 1:**
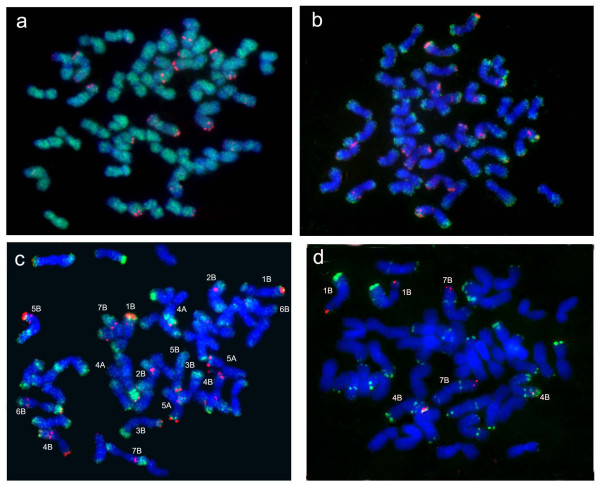
**FISH to mitotic metaphase chromosomes of *T. aestivum***. The cultivars analyzed are (a, b) *T. aestivum *cv. Chinese Spring and (c, d) *T. aestivum *cv. Renan. Probes were labeled with biotin and digoxygenin, detected with avidin-FITC (green) and anti-digoxygenin-rhodamine (red), respectively. The yellow signal arises from colocalized probes. The probe combinations: (a) BAC_2322J20 (green) and pSc119 (red); (b) BAC fragment (2322J20/30) (green) and pSc119 (red); (c) BAC_2050O8 (green) and pSc119 (red); and (d) Spelt52_2050O8 (red) and pSc119 (green).

The results were summarized in Table 1. The size of the inserted fragments varied from 87 to 168 kb. The comparison of the chosen BAC clones based on the length of fragments obtained by *Not*I, *Hin*dIII, *Bam*HI, and *Eco*RI hydrolysis showed that the cloned DNA could partly overlap in the following BACs: (1) 2322J20 and 2424P01, (2) 110O07 and 1487N20. High content of repetitive DNA sequences was a characteristic feature of all the chosen clones. According to Southern hybridization of *Hin*dIII, *Bam*HI, *Eco*RI fragments of BAC clones to common wheat total DNA, the highest content of repeats was 80% and the lowest, 25%. The results of *in situ *hybridization were consistent, confirming a high content of repeats (mainly of mobile elements) in BAC clones. The hybridization signals covered the entire length of *T. aestivum *chromosomes for eight of the nine BAC clones (Figure 1a); of these eight clones, two BAC clones (2383A24 and 112D20) hybridized to a limited set of chromosomes. Predominantly subtelomeric location on the common wheat chromosomes was demonstrated for BAC_2050O8 only; in this clone, the repeat content was lower then in other clones (Figure 1c, Table 1). The distribution pattern of BAC_2050O8 on the chromosomes was different. Some of the chromosomes, such as 5A, gave a distinct hybridization signal only at the chromosomal end, whereas in other chromosomes, such as 4AL, 5BL, and 7BL, hybridization signal displayed multiple locations on their distal third. Weak signals in the interstitial regions of chromosomes indicated that part of the sequences present in this BAC clone were present not only in telomeres, but also in other chromosome regions. The Spelt1 and Spelt52 contents in the selected BAC clones were estimated by dot hybridization. The BAC clones isolated by PCR using Spelt52(F/R) primers contained 23-28 copies of Spelt52. No Spelt1 repeats were found in these clones (Table 1). The BAC clones selected by the hybridization to the Spelt1 probe contained up to seven copies of this repeat. Among them, Spelt1 was detectable only in single copies in clone 112D20. The Spelt52 sequences were also identified in clone BAC_112D20, which was originally selected using the Spelt1 probe (Table 1). To confirm that Spelt1 and Spelt52 were organized in tandem arrays in the corresponding BAC clones, we assayed the corresponding BAC clones using two methods: PCR with the primers specific to these two repeats and RFLP analysis of partially digested BAC clones (*Hae*III for Spelt1 and *Eco*RI for Spelt52). The arrays of tandemly arranged Spelt1 sequence were found in BAC clones 2322J20, 2383A24, and 2424P01 and arrays of Spelt52, in BAC clones 110B21, 2050O8, 478L20, 110O07, and 1487N20 (data not shown). The BAC clones were also examined for the presence of the tandem repeats pSc119.2 and pAs1, used for identification of *T. aestivum *chromosomes. Part of these repeats was located in the subtelomeric regions in the common wheat chromosomes [29]. Southern hybridization showed that pSc119.2 repeats were absent in these BAC clones, whereas pAs1 repeats were present in BAC_478L20 only, giving a rather strong hybridization signal (Table 1). No telomeric repeats (TTTAGGG)n were identified in all BAC clones when using telomeric repeat synthetic probe (see Materials and Methods).

### *In situ *hybridization of BAC fragments

It is very difficult to accurately localize wheat BAC clones on chromosomes using *in situ *hybridization, because they are very rich in repetitive DNA sequences, mainly transposons, present everywhere on the chromosomes [30]. Thus, we attempted to localize these BAC clones through their fragmentation and *in situ *hybridization of the derived fragments on the chromosomes of common wheat. The procedure consisted in (1) choosing and hybridization of BAC fragments covering more than 10 kb and free of repeats or repeat-poor and (2) hybridization of the fragments containing Spelt52 sequences. The repeat-poor BAC fragments were chosen based on hybridization of BAC restriction fragments with total wheat DNA and with the Spelt1 and Spelt52 probes. The DNA fragments of 10 kb and longer were used as probes for *in situ *hybridization. Consequently, we succeeded in isolating fragments of about 30 kb (2322J20/30) and 40 kb (478L20/40) from BAC clones 2322J20 and 478L20, respectively. These two fragments were localized to the subtelomeric chromosome regions; the result of *in situ *hybridization of 2322J20/30 is shown in Figure 1b. A fragment of about 21 kb was isolated from BAC_2050O8. *In situ *hybridization of this fragment confirmed its subtelomeric localization; however, this approach failed to identify precise chromosome localizations of BAC_2050O8. For the remaining BAC clones, the attempts to identify repeat-poor fragments were unsuccessful, presumably, because of the abundance of repetitive sequences (Table 1). The presence of Spelt52 tandem repeats with the total length of 8500-10 000 bp (the monomeric unit of 380-390 bp) in the BAC clones of cv. Renan made this repeat advantageous as a probe for localizing BACs (Table 1). With this in mind, we performed *in situ *hybridization of a PCR fragment of BAC_2050O8 (Spelt52_2050O8), obtained using Spelt52(F/R). Spelt52_2050O8 hybridized to the ends of three chromosome arms: 1BS, 4BL, and 7BL (Figure 1d).

### Analysis of nucleotide sequence of subtelomeric BAC_2050O8

**Figure 2:**
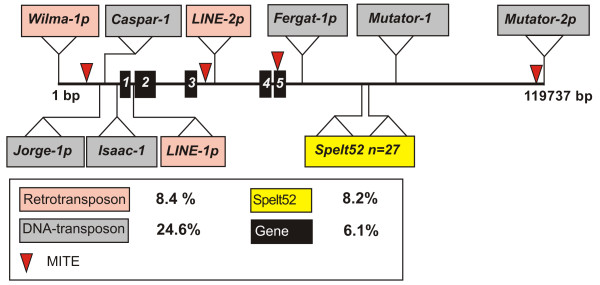
**Structural organization of *T. aestivum *genomic stretch of 119 737 bp tagged by Spelt52 subtelomeric repeats**. In the names of transposable elements, "p" at the end means a partial element with deletions at the ends.

For a more detailed analysis of the structure of subtelomeric DNA, the BAC_2050O8 with a predominant subtelomeric *in situ *localization was completely sequenced according to Chantret et al. [31]. The obtained sequence was annotated according to the following strategy. First, the repetitive sequences were identified in the Triticeae Repetitive Sequence Database (TREP) [32] using the BLASTN and BLASTX algorithms [33] and in the RepBase [34] using the CENSOR program [35]. Then, we predicted the genes using the GeneMark.hmm [36] and FGENESH programs [37]. The structure of the ~120 kb BAC clone is shown in Figure 2. The transposable elements identified in 2050O8 were listed in Table 2. All the repetitive sequences (including transposons and tandem repeats) in this BAC clone constituted 48.9%. Interestingly, DNA transposons were predominant, representing 24.6% of the entire BAC clone length, whereas retrotransposons constituted only 8.4%. The main DNA transposon subclass in subtelomeric BAC_2050O8 was CACTA, constituting 53.1% of all transposable elements and 17.5% of the BAC clone length. CACTA was represented by *Caspar *and *Isaac *elements with complete ends and partial *Jorge *and *Fergat *elements (Table 2). *Caspar*_2050O8 was 11 666 bp in length; in addition, it was the only full-length element, which contained two ORFs (open reading frames) corresponding to the transposase and CTG-2 genes. The Spelt52 sequence represented 8.2% of the total BAC clone length (Table 2). Sequence analysis confirmed that aside from Spelt52, no other tandem repeats were identified. Five hypothetical genes were identified and accounted for 6.1% of the total BAC clone length. Analysis of the predicted ORFs, which were not the parts of transposable elements, was detailed in Table 3. The first two genes were homologous to the hypothetical rice genes and contained conserved xyloglucan endotransglycosylase and riboflavin deaminase-reductase domains; the third was a putative gene for peroxisomal membrane protein PEX11-1. The remaining two ORFs were hypothetical genes: one contained the armadillo/beta-catenin-like repeats, and the other was unknown, predicted by FGENESH [37] and GeneMark.Hmm [36]. The genes were separated by LINE retrotransposon insertion and unassigned DNA sequences. BLAST alignments of the BAC_2050O8 sequence and the contigs containing mapped wheat ESTs (expressed sequence tags) from GrainGenes database [38] identified two contigs, 1802 and 11876, with a high homology to 2050O8 sequence. EST contig 1802 was located on chromosomes 6AS and 6BL [, NSFT03P2_Contig1802]. The level of homology between EST consensus 1802 (length 489 bp, location on 6AS and 6BL) and BAC 2050O8 sequence (length 501 bp, position 98604-99104 bp) was 86%. The corresponding region of 2050O8 was annotated as unassigned DNA sequences. The homology of the other EST contig 11876 [, NSFT03P2_Contig11876] (length 826 bp, location on 5AL, 4BL, and 4DL) to BAC_2050O8 was higher (97%) in a 805 bp stretch, and the region of homology was in the region of the putative peroxisomal membrane protein PEX11-1 (Table 3). It was of interest that comparison of the exon-intron structure between PEX11-1_2050O8 and EST contig revealed a complete correspondence of the EST to the coding part of the gene. Comparison of restriction sites in BAC_2050O8 with the mapping data for EST contig 11876  suggested that BAC_2050O8 was localized to the telomeric part of chromosome 4BL (bin 4BL-5 0.86-1.0). BAC_2050O8 could be more precisely localized according to the homology with EST sequence BE638121 from contig 11876, which was mapped to the telomeric bin 4BL-10 0.95-1.0 [39].

**Table 2:** List of transposable elements identified in BAC clone 2050O8.

**Class, subclass, family**	**Copy number**	**Length, bp**	**%**
**Class I elements (retrotransposons)**		**10 100**	**8.4**

LTR-retrotransposons (*Gypsy*-like, *Wilma*)	1	2577	2.2

Non-LTR retrotransposons (LINE)	2	5756	4.8

Inverted repeats similar to *O. sativa *LINE reverse transcriptase	2	1767	1.4

**Class II elements (DNA transposons)**		**29 053**	**24.6**

CACTA	4	20 962	17.5
*Caspar*	1	11 666	
*Isaac*	1	5817	
*Jorge*	1	2210	
*Fergat*	1	1269	

Mutator	2	7674	6.4

Similar to Harbinger transposase	1	417	0.4

MITE	4	319	0.3

**Other known repeats**		**19 019**	**15.9**

Spelt52 tandem repeats	27	9825	8.2

Other tandem repeats (monomer of > 80 bp)		1526	1.3

SSRs		351	0.3

GSS + STS		869	0.7

Direct repeats (> 100 bp)		509	0.4

Region of homology to			
AY485644		4374	3.7

AY951944		1565	1.3

**Genes**	**5**	**7257**	**6.1**

**ESTs**		**8559**	**7.1**

**Unassigned sequence**		**45 430**	**37.9**

Positions of the elements in BAC clone are indicated; SSRs, simple sequence repeats; GSS (genome survey sequences) + STS (sequence-tagged sites); and ESTs, expressed sequence tags.

**Table 3:** List of genes predicted in BAC clone 2050O8.

**Number** **in Figure 2**	**Function**	**Position**	**Length**	**Note**
1	Conserved hypothetical protein containing xyloglucan endotransglycosylase domain	30 566- 31 296	235 aa 731 bp	Similar to OsJ_008848, Os03g02610 EST: +

2	Conserved hypothetical protein containing riboflavin deaminase-reductase and pyrimidine reductase domains	35 299- 37 418	396 aa 2120 bp	Similar to OsI_009521, Os03g02600 EST: +

3	Putative peroxisomal membrane protein PEX11-1	41 950- 43 275	237 aa 1326 bp	Similar to Os03g0117100, Os03g02590 EST:+

4	Conserved hypothetical protein containing armadillo/beta-catenin-like repeats	58 480- 60 177	565 aa 1698 bp	Similar to OsI_009519, Os03g02580 EST:+

5	Unknown, predicted by FGENESH and GeneMark.hmm	60 788- 62 170	273 aa 1383 bp	No significant similarity to proteins and ESTs

We performed the BLAST search for the predicted genes in BAC_2050O8 and their protein products in the Rice Genome Annotation Project Database [40]. We found that the four predicted wheat genes, displaying a significant homology to four putative rice genes, belonged to clone OSJNBa0056G13 pseudomolecule (virtual contig), located at the distal end of rice chromosome 3S [, GenBank:AC134236] (Table 3). The rice genes were located close to each other, and their order was the same as that of the corresponding genes in BAC_2050O8; however, one of the genes, namely, Os03g02610, was in the opposite orientation as compared with BAC_2050O8. All wheat genes showed a significant homology at the amino acid and nucleotide levels to the putative expressed rice genes. At the amino acid level, gene_2050O8-3 matched best to the rice putative protein Os03g02590 (peroxisomal membrane protein PEX11-1), displaying 87% homology over the entire length. Both these genes (rice and wheat) had six introns of about 100 bp. The identity of coding sequences is 81%; 71% of the nucleotide changes were synonymous. The identity of introns is 37-47%.

Other genes also demonstrated significant homology to rice (E < e-80 for protein): at the amino acid level, gene_2050O8-1 was homologous to Os03g02610 (xyloglucan endotransglycosylase/hydrolase protein 5 precursor), gene_2050O8-2 to Os03g02600 (riboflavin biosynthesis protein ribD), and gene_2050O8-4 to Os03g02580 (armadillo/beta-catenin-like repeat family protein) (Table 3).

### Analysis of Spelt52 nucleotide sequences in BAC_2050O8

The Spelt52 sequences in BAC_2050O8 were organized as two arrays of repetitive units with a "head-to-tail" orientation. The first array consisted of seven complete units and one truncated fragment at its 3' end. The second array was located 1375 bp from the first and consisted of 18 complete and two outermost truncated units.

To estimate the degree of Spelt52 sequence conservation in BAC_2050O8, we aligned the nucleotide sequences of 25 complete units using the Multalin program [41] and calculated for each monomer the percentage of identity to the consensus sequence obtained from the Multalin alignment [see Additional file 1]. The mean identity to the consensus was 93% (within the range of 88-96%). There were no specific differences between the elements from the first and second arrays.

To investigate the evolution of Spelt52 repeat in Triticeae species, all the publicly available Spelt52 sequences were used. Five Spelt52 sequences [GenBank: AY117400, AY117401, AY082346, AY082347, Z21644] were found, all derived from *Ae. speltoides *genome. It was earlier demonstrated that the Spelt52 repeat family consisted of two types of monomeric units: Spelt52.1 and Spelt52.2, which shared a conserved 280 bp region and had short (110 and 96 bp) variable regions [26]. AY117400 and AY117401 were the Spelt52.1 and Spelt52.2 monomers, respectively, whereas clones AY082346, AY082347, and Z21644 consisted of two Spelt52.1 and Spelt52.2 monomeric units.

**Figure 3:**
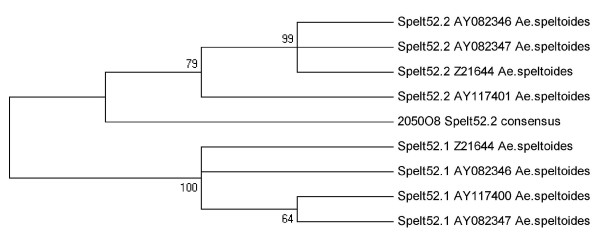
**Phylogenetic tree of the Spelt52.1 and Spelt52.2 sequences from *T. aestivum *and *Ae. speltoides***. *Ae. speltoides *sequences Spelt52.1 and Spelt52.2 were taken from GenBank. All Spelt52 sequences contained in *T. aestivum *BAC_2050O8 were represented by the consensus Spelt52_2050O8 sequence. Neighbor-joining phylogenetic tree with 500 bootstrap replicates was constructed using MEGA4 software package [42].

We performed phylogenetic analysis of all the repeat monomers that contained conserved and variable regions. For Spelt52 sequences from BAC_2050O8, we took the consensus of 25 complete monomeric units. We considered the consensus as an adequate representative of the bulk of Spelt52_2050O8 sequences, because the monomer sequences displayed no specific differences. The phylogenetic tree constructed using MEGA4 [42] contained two major branches formed by Spelt52.1 and Spelt52.2 sequences. The *T. aestivum *consensus clustered with the Spelt52.2 sequences of *Ae. speltoides*, indicating that BAC_2050O8 contained only the monomers of Spelt52.2 subfamily (Figure 3).

## Discussion

### Spelt1 and Spelt52 as probes for isolating subtelomeric clones

The gaps remaining in the telomeric regions pose hindrances to complete sequencing of eukaryotic genomes [22]. New genomic libraries were constructed using various plasmid vectors for closing gaps and capturing the telomeric sequences [23,43]. The diversity of DNA that marks the telomeric/subtelomeric regions facilitates the task of sequencing regions at the chromosomal ends. This is quite timely in view of the wheat genome sequencing initiative [44]. Our relevant finding was that Spelt1 and Spelt52 sequences were specific markers of the chromosome ends of *Ae. speltoides *and several polyploid wheat species. Hybridization of Spelt52_2050O8 probe to the wheat cv. Renan chromosomes detected three sites with a weak signal per haploid genome at chromosome ends (Figure 1d). Spelt1 was localized on two chromosome ends per haploid genome of cv. Renan; one site gave a rather strong hybridization signal (data not shown). Use of Spelt1 and Spelt52 for screening the BAC library of cv. Renan enabled us to choose nine clones presumably associated with the subtelomeric regions (Table 1). BAC_2050O8 was localized by *in situ *hybridization (at the levels of both the clone and subclones) predominantly to the subtelomeric chromosome regions (Figures 1c, d). Comparing the *in situ *hybridization data for the Spelt52_2050O8 and the BLAST search against the wheat mapped EST contigs [38], we localized 2050O8 to the end of 4BL. A subtelomeric localization of two additional BAC clones, 2322J20 and 478L20, was demonstrated by *in situ *hybridization of their subclones 2322J20/30 and 478L20/40, respectively (Figure 1b). Since the Spelt52 probe hybridizes only with the subtelomeric regions of chromosomes 1BS, 4BL, or 7BL, we can assume that the BAC clones containing over 23 copies (8740 bp) of the Spelt52 repeats were from the subtelomeric regions of chromosomes 1BS, 4BL, or 7BL (Table 1). Of the Spelt1-containing BAC clones, 2424P01, partly overlapping with clone 2322J20, can be also ascribed to subtelomeric regions (Figure 1b). The *in situ *hybridization on the chromosomes of cv. Renan detected two terminal blocks per haploid genome. However, the screening of BAC library with Spelt1 probe found no clones containing Spelt1 regions with a length exceeding 5000 bp (the region of repeated DNA detected by *in situ *hybridization) (Table 1). Two reasons can explain the absence of extended Spelt1 regions in the selected clones, namely:

(1) The terminal chromosome regions, containing longer Spelt1 repetitive sequences, escaped cloning when obtaining the genomic BAC library of *T. aestivum *cv. Renan, as they could be cut off by the restriction endonucleases *EcoR*I, *Hin*dIII, and *BamH*I, used for hydrolysis [24]; and

(2) A high recombination activity in the region of Spelt1 repeats could result in discharge of part of these repeats during construction of this library. Such effects were observed earlier when we attempted to clone the tandem Spelt1 repeats with a length of 2000 bp and more (unpublished data). A high recombination level leading to deletions of Spelt1 repeats in the progeny of individual self-pollinated plants was also observed [45].

A subtelomeric localization of two clones, 2383A24 and 112D20, was questionable because of a low Spelt1 and Spelt52 copy number and a failure of *in situ *hybridization approach in determining their precise localization. Thus, the example of Spelt1 and Spelt52 repeats demonstrated that satellite DNA sequences from the subtelomeric regions of diploid wheat progenitor can be used for selecting the BAC clones from the corresponding regions of common wheat chromosomes.

### Specific features in organization and evolution of Spelt52 repeats

The Spelt52 repeats were undetectable in polyploid Emmer wheats (BBAA and BBAADD genomes) by *in situ *hybridization [12,25]. On the other hand, both blot hybridization and PCR indicated the presence of individual sequences belonging to this family in some studied accessions of the polyploid wheats with the BBAA and BBAADD genomes [46]. The failure of *in situ *hybridization in detecting these sequences can result not only from that detection of the DNA sequences shorter than 5000 bp by this method is hindered, but also with the divergence between the probe used for hybridization and the homologous sequences within the studied species. In particular, it has been shown that two probes with a homology of 87.3% give quite different hybridization patterns of the tandem repeat family pAs1, found in the genomes of *Ae. tauschii *and barley [47].

Use of the primers for Spelt52 repeat allowed us to detect a tandem organization of several BAC clones already when screening the BAC library. The amplification of Spelt52 fragments using the same primers and BAC_2050O8 as a template and produced probe for hybridization allowed us for the first time to localize Spelt52 repeats in the chromosomes of common wheat by *in situ *hybridization to the ends of chromosome arms 1BS, 4BL, and 7BL of wheat cv. Renan. Note that the PCR fragment of Spelt52 sequence was successfully mapped earlier to the end of the genetic map of 4BL chromosomes [39].

The 25 Spelt52 sequences detected in BAC_2050O8 displayed a high level of homology (on the average, 93%). Although BAC_2050O8 contains two regions with Spelt52 tandems, the clustering of repeated units into two groups according to the primary structure was not observed. It is impossible to compare it with extended Spelt52 tandem regions of other accessions and species as the corresponding tandem regions are absent in the relevant databases. Spelt52 family contained two types of repeated units -- Spelt52.1 and Spelt52.2; note that these monomers in the majority of the studied accessions *Ae. speltoides *alternate [26]. The tandems formed by Spelt52.2 sequences have been found only in one *Ae. speltoides *accession, TS01. In the hexaploid wheat genome, in BAC_2050O8, we detected only Spelt52.2 tandem sequences (Figure 3). Taking into account that *Ae. speltoides *is the most likely donor of the B/G genomes of polyploid wheats, it would be useful to include the presence of different variants of the tandem arrangement of Spelt52 in the phylogenetic studies of *Sitopsis *and *Triticum *species.

A characteristic feature of the tandem repeats is their ability to amplify during the evolution, thereby increasing their copy numbers and creating a high level of intraspecies polymorphism in the copy number [48]. An illustrative example of such variation is the Spelt1 family with the repetitive unit of 178 bp. The number of sites where this repeat is localized varies in a wide range in both *Ae. speltoides*, for which it is genome-specific, and in polyploid species, to whose formation *Ae. speltoides *has contributed [12]. Both individual plants of the same accession and the progeny of the same plant can differ in the copy number of this repeat [45]. The level of Spelt52 polymorphism is also rather high [12].

The amplification of the same tandem subtelomeric repetitive DNA sequences in evolutionary remote cereal species has been described, whereas such amplification is absent in closely related species. For example, the repeat family 350 bp/pSc200/pSc74 is characteristic of *Secale cereale, S. montanum, Dasypyrum villosum, D. breviaristatum*, and *Agropyron cristatum; *however, it has not been detected by hybridization in other rye species [49]. Spelt52 repeats have been localized to chromosomes of *Ae. speltoides *and other two from four *Sitopsis *species (*Ae. longissima *and *Ae. sharonensis*) [12,25]. The diversity on the presence of tandem repeats in the subtelomeric chromosome regions of various cereal species suggested the existence of a common pool of sequences able to amplify during evolution, forming a subtelomeric repeat family. Cloning and PCR analysis of the amplification product obtained from the rice (belonging to other cereal tribe) genome using the primers specific to Spelt1 and Spelt52, we detected no homology to either Spelt1 or Spelt52 repeats except for the primer sequences (EA Salina, unpublished data). We have not also found any degenerate sequences or sequences homologous to Spelt52 repeat when comparing wheat syntenic BAC_2050O8 and rice OSJNBa0056G13 pseudomolecule. Presumably, the pool of the common sequences involved in amplification events during evolution exists yet is stringently confined to the frame of a taxonomic unit. Most likely, such unit for cereals is a tribe.

### Microcollinearity in the region of BAC_2050O8 of wheat chromosomes 4BL and rice chromosome 3S

According to the data on distribution of EST DNA sequences, the wheat group 4 chromosomes display the most pronounced similarity to the short arm of rice chromosome 3 [39,50,51]. Individual deviations from collinearity were observed in the centromeric regions and distal part of the consensus chromosome 4BL map. The distal region of wheat chromosome 4BL has been compared in detail with rice pseudochromosome 3S [39]. The most pronounced distinctions were recorded in the terminal telomeric bin 4BL-10 (0.95-1.0), which displayed a higher recombination level. BAC_2050O8, which contains a sequence with a 90% homology to EST BE638121, is located at a distance of 17 mapped ESTs from the 4BL chromosome end (about two-thirds of the entire terminal bin) [39]. The four putative rice genes, belonging to OSJNBa0056G13 pseudomolecule and displaying a significant homology to four predicted wheat gene in BAC_2050O8, are located at a distance of about 870 kb from the end of rice chromosome 3S. The terminal regions of wheat (BAC_2050O8, chromosome end) and rice (OSJNBa0056G13, chromosome end) displayed the following differences: (1) 10 of the 17 wheat ESTs located in this region have no homologous sequences in rice, and (2) the DNA sequences covering about 500 kbp from the end of chromosome 3S have no homologs on chromosome 4BL (according to BLAST results on February 20, 2009). In addition, comparison of the order of wheat ESTs and rice pseudomolecules suggested that at least two inversion events have occurred in this chromosome region besides duplications (data not shown). The changes in orientation of the first of the four genes predicted in BAC_2050O8 (encoding a hypothetical protein with xyloglucan endotransglycosylase domain) relative to the corresponding rice genes can result from such inversions. Note that the rice region (in OSJNBa0056G13) containing these four genes is of 9556 bp versus 29 612 bp of the corresponding wheat region in BAC_2050O8 (Table 3). This difference results from an enlarged intergenic region in BAC_2050O8 (totally amounting to 23737 bp versus 3641 bp in rice). The intergenic regions in BAC_2050O8 contains two degenerated LINE elements (2941 and 2815 bp), one MITE element (117 bp), and an unassigned sequence. At the nucleotide level, the intergenic regions of rice and wheat showed no similarity to each other, and the regions flanking this block of genes in rice showed no similarity to the overall BAC_2050O8 sequence.

The wheat regions containing the four predicted genes in BAC_2050O8 is more than threefold longer that the corresponding region from rice OSJNBa0056G13, whereas the overall wheat genome exceeds the rice genome more than 40-fold and the distal region of chromosome 4BL is 30-fold longer than the corresponding homologous region in rice [39]. Thus, it is most likely that the wheat genome increased in the regions free of genes. In the case of BAC_2050O8, these are the regions of retrotransposons, CACTA transposons, Spelt52 tandem repeats, and unassigned sequences.

## Conclusion

Analysis of the *T. aestivum *cv. Renan BAC clones tagged by subtelomere-specific sequences Spelt1 and Spelt52 allowed us to demonstrate the utility of Spelt1 and Spelt52 subtelomeric repeats for isolation of the BAC clones carrying subtelomeric DNA from genomic libraries. Analysis of the entire 119 737 bp nucleotide sequence of BAC_2050O8, the subtelomeric localization of which was demonstrated by FISH, provided new data on the primary structure of one of the distal regions in the *T. aestivum *B genome. In BAC_2050O8, class II transposons are predominant and constitute 24.6% of the entire clone length, while class I transposons account only for 8.4%, although the latter transposon class is prevalent in majority of the other published *Triticeae *BAC clones [52-54]. Among class II transposons, the most abundant is CACTA *Caspar*_2050O8.

The profound analysis of BAC_2050O8 sequence together with FISH of Spelt52 sequences derived from BAC_2050O8 allowed us to localize it to the terminal bin 4BL-10 (0.95-1.0). A microcollinearity of the wheat (BAC_2050O8, 4BL) and rice (OSJNBa0056G13, 3S) gene regions, encoding three hypothetical proteins and putative peroxisomal membrane protein PEX11-1, was demonstrated. It was shown for the first time that Spelt52 sequences have been involved in the evolution of the terminal regions in hexaploid wheat chromosomes.

## Methods

### BAC library screening

The BAC clones carrying subtelomeric tandem repeats Spelt1 and Spelt52 [26] were obtained from the genomic BAC library of *T. aestivum *cv. Renan [24] by hybridization with Spelt1 probe and PCR screening using the primers specific to Spelt52.

### Pulsed-field gel electrophoresis (PFGE)

BAC DNA preparation was obtained with a modified alkaline lysis procedure [55]. To estimate the insert length of BAC clones, BAC plasmid DNA (2 μg) were digested with *Not*I to release the insert from the vector. Restriction fragments were separated in 1% (w/v) agarose gel (14°C, 15 h, 150 V, ramped from 1 to 15 seconds) using a contour-clamped homogeneous electric field (CHEF) [56]. The electrophoresis buffer was 0.5 × TBE (45 mM Tris-borate pH 8.0 and 1 mM EDTA). Lambda concatemer was used as a molecular size standard. The lengths of restriction fragments were measured using the Gel analysis v1.0 program (Lytech, Russia)

### PCR analysis

The specific primers were used for Spelt1 (Sp1F, 5'-tccaa-accat-ccccg-tcaag-cg-3' and Sp1R, 5'-aagtt-cttct-ggccg-tgcca-ta-3') and Spelt52 (Sp52F, 5'-gcaca-caaac-ccgga-gaaag-t-3'and Sp52R, 5'-tcccc-gtttc-ttctc-tagcc-t-3') [26] repeats. PCR analysis of BAC clones was performed in an Eppendorf Mastercycler according to the following mode: 30 cycles of 1 min at 94°C, 1 min at 55°C, and 2 min at 72°C followed by a final stage of 15 min at 72°C. PCR products were separated by electrophoresis in 1% agarose gel.

### Southern blot hybridization

Plasmids with inserted telomere-associated tandem sequences Spelt1 and Spelt52, isolated from *Ae. speltoides*; pSc119.2 from *S. cereale*; and pAs1 from *Ae. tauschii *were used as probes [14,26-28]. Telomeric repeat synthetic probe was obtained by PCR according to the protocol originally described by [57]. Spelt1, Spelt52, pSc119.2, and pAs1 probes were labeled by the random hexamer method with α-^32^P-dATP (Amersham Pharmacia Biotech, United Kingdom) using Klenow fragment [58]. BAC DNA (2 μg) were digested by *Hin*dIII, *Bam*HI and *Eco*RI restriction endonucleases, or partially digested with *Hae*III (for Spelt1) and *Eco*RI (for Spelt52), separated in 1-1.2% agarose gel, and transferred to a Hybond-N+ membrane (Amersham Pharmacia Biotech, United Kingdom). Filters were first moistened by floating in 2 × SSC. Pre-hybridization was carried out at 65°C for 4 h in 6 × SSC, 5 × Denhardt's solution, 0.5% SDS, and denatured salmon sperm DNA (100 mg/ml). Hybridization was conducted at 65°C for 16 h in the same solution with denatured labeled probe (1 ng/ml). After hybridization, filters were washed at a room temperature in 2 × SSC, 0.1% SDS; 0.5 × SSC, 0.1% SDS and 0.1 × SSC, 0.1% SDS for 15 min each. The membranes were exposed with Kodak X-ray film for a few hours to 3-7 days at -70°C depending on signal intensity.

### Dot blot hybridization

The copy number of Spelt1 and Spelt52 sequences in BAC clones was estimated by dot blot assay [59]. The serial dilutions (1, 0.5, 0.1, and 0.05 μg) of each BAC DNA were spotted onto a Hybond N+ membrane. The plasmid DNA with Spelt1 and Spelt52 inserts were used as a standard with a copy number of 10^8 ^to 10^11^. BAC and plasmid DNA concentrations were determined by spectrophotometry and gel electrophoresis. The DNA samples were denatured with 0.3 M NaOH for 20 min before spotting, neutralized after spotting with 2 × SSC for 5 min, cross-linked to membrane using UV light, dried, and used for dot blot hybridization. The conditions of pre-hybridization and hybridization were as described above for Southern blot hybridization. The signal intensity was counted for each spot by a MiniBeta (LKB). The mean number of counts of the α-^32^P-dATP-labeled Spelt1 and Spelt52 probes was calculated for each BAC. The copy number of specific sequences in BAC DNA was estimated by comparing the hybridization intensity with the standard.

### Fluorescence in situ hybridization (FISH)

For fluorescence *in situ *hybridization, we used BAC clones, recombinant plasmids, synthetic telomeric probes, or large specific DNA fragments. Large specific DNA fragments (more than 10 kbp long and containing no repetitive sequences) were isolated from agarose gel according to [55]. BAC DNA was treated with restriction endonucleases *Bam*HI, *Hin*dIII, and *Eco*RI, ran through agarose gel until fragments separated well, transferred to Hybond N+ membrane, and hybridized with α-^32^P-dATP-labeled total DNA of *T. aestivum *cv. Chinese Spring. The bands that showed no hybridization signals and were longer than 10 kbp were isolated and labeled. DNA probe (1 μg) was labeled by nick translation to incorporate biotin-16-dATP (Life Technologies, Gibco BRL) or digoxigenin-11-dUTP (Boehringer Mannheim) according to the manufacturers' protocol. Telomeric probe was labeled by PCR to incorporate biotin-16-dATP (Life Technologies, Gibco BRL). Metaphase chromosomes were prepared and FISH was performed according to [12] with minor modifications. To identify chromosomes carrying signals, we used the probe combinations pSc119.2 + BAC and pAs1 + BAC. The chromosomes were identified according to standard chromosome nomenclature [60,61].

### BAC sequencing and sequence analysis

BAC shotgun sequencing and sequence assembly were carried out at the National Center of Sequencing (Evry, France) as described [31]. Sequence annotation was done according to the Guidelines for Annotating Wheat Genomic Sequences from the International Genome Wheat Sequencing Consortium [44]. The procedure was two-step, including identification of repetitive elements and identification of gene structure. For identification of DNA repetitive elements, we screened the Triticeae Repetitive Elements Database at GrainGenes (TREP) [32], Rice Genome Annotation Project Database [40], NCBI non-redundant nucleotide databases [62] using the BLASTN algorithm [33] with a cutoff value of 1e^-5^, and RepBase [34] using CENSOR program [35]. Hypothetical transposable element proteins were identified by BLASTX algorithm against TREP hypothetical protein and NCBI non-redundant protein databases. Gene structures in BAC_2050O8 were identified by integrating the results of predictor programs GeneMark.hmm [36] and FGENESH [37]. The putative functions were assigned through BLASTN searches against NCBI EST database (dbEST), BLASTP, and BLASTX searches against NCBI non-redundant protein and Rice Genome Annotation Project database [40] according to criteria described in the Guidelines for Annotating Wheat Genomic Sequences [44]. The DNA sequences that were not assigned to transposable elements or genes were considered as unassigned DNA. The alignments of Spelt52 nucleotide sequences were performed by Multalin program [41]; phylogenetic trees were constructed using a neighbor-joining method by MEGA4 software package [42]. The nucleotide sequence reported in this paper is deposited in the GenBank database under accession number GQ165812.

## Abbreviations

(BAC): Bacterial artificial chromosome; (TE): transposable elements; (FISH): fluorescence *in situ *hybridization; (ESTs): expressed sequence tags;

## Authors' contributions

EMS, ABS and EAS carried out the molecular genetic studies and data analysis. IGA performed FISH analysis. HB and CH carried out the BAC clone sequencing. DAA participated in nucleotide sequence annotation. EAS drafted and edited the manuscript. BC conducted the coordination of BAC analysis and the manuscript conception. All authors read and approved the final manuscript.

## Supplementary Material

Additional file 1 **Alignment of the DNA sequences of 25 Spelt52.2 units contained in BAC_2050O8**. The consensus sequence for these Spelt52.2 units is derived by Multalin program [41]. The conserved positions in consensus were shown with capital letters and the variable, with lower-case letters. The 25 Spelt52.2 units were aligned; the conserved nucleotide positions are indicated by dot, the variable by letters, and the gaps by dashes. For each unit, the sequence length and homology to consensus are specified. Click here for file
