# Supplementary material for: Isolation and sequence analysis of the wheat B genome subtelomeric DNA
Source: BMC Genomics. 2009 Sep 5;10:414. doi: 10.1186/1471-2164-10-414 (PMC2756281; doi:10.1186/1471-2164-10-414)
Supplement: Additional file 1 — Alignment of the DNA sequences of 25 Spelt52.2 units contained in BAC_2050O8. The consensus sequence for these Spelt52.2 units is derived by Multalin program [41]. The conserved positions in consensus were shown with capital letters and the variable, with lower-case letters. The 25 Spelt52.2 units were aligned; the conserved nucleotide positions are indicated by dot, the variable by letters, and the gaps by dashes. For each unit, the sequence length and homology to consensus are specified. [file 1471-2164-10-414-S1.pdf]

```
                  1                                                                                               100
205008_1-1        .......C............................................................C.........G.............T.......
205008_1-2        .......C.......G..A....................................A............T.........G....T................
205008_1-5        ...C...C............................C..........C.....T.............AT.........G..........A..........
205008_2-19       .......G...........T......T....T................................G...C.........G.....................
205008_2-17       .......C...........................T...T...........T................T.........G.....................
205008_2-8        .......C.........................................C..TT......A.......T.........G.....................
205008_2-5        .......C...T...............................G........................C.........A.....................
205008_2-11       .......C...T...............................G........................C.........A ....................
205008_2-14       .......T...T...........A............................................C.........A.....................
205008_2-4        .......C...................................T.......................AC.........G.....................
205008_1-3        .......C...................A................A...........C...........CA........A.....................
205008_1-6        .......C...T........T.T....G................A...........C......C....C......T..A.....................
205008_2-12       .......C.............................................T.......-......T.........G.....................
205008_2-9        .......C..............T...................T..........T..............T.........G.....................
205008_2-7        .......C...............................A....................C..A....T.........G.G...... ........A...
205008_2-15       ....A..A.........A...................C.A.......................A....T.........A........ ............
205008_2-18       C......C...............AC..........A............-...................T.........G.....................
205008_2-6        .......T............-.T........................G...T................CA........G..T..................
205008_2-16       .......C...........................-................................T....C....G.......A.....T......A
205008_1-4        .......C............................................................T.........A.....................
205008_1-7        .......C............................................................T.........G.....................
205008_2-10       .......T...T................................A.......T...............T.........AT...........T........
205008_2-13       .......T...............A..................T...........A.............T.........G.....................
205008_2-2        .......C...............................T............T..A............T.........A.....................
205008_2-3        .......C...........................................T...A............C.....T...A.............T.......
CONSENSUS         GTTAGTTcTGAgTGAATTCCCTcgGTATGATCATTcTTTcATCagTTTGTTcccCgTTTCTTCtCTAGtCTGAGCCTAgCACACTATTCTAGcTATCCAT
                   **  ** *** *** *    *   *  *** ***   * **   **   *      ***   *  **   ***   **    * **  * *  *** **

                  101                                                                                             200
205008_1-1        .......--...........C....A.T..............G..A................A....T..........A.............G.......
205008_1-2        .......--....A......C.....................G........................T.T......................G......G
205008_1-5        .......--.GC........C....A................G.......A....G...........A.............G..........T.....T.
205008_2-19       .......--....A......C................T....G....G...................T........................A.......
205008_2-17       .......--...........C..........T.........AG...T.........A..........T......TT..............T.A......A
205008_2-8        .T.....TG...T.......C.....................G........................A.T...........C.........CG.......
205008_2-5        .......--...........C...........T.........G.................T..G...T........................G.......
205008_2-11       .......--......T....C.A...................G........................G.....C........G.........G.......
205008_2-14       .......--...........C.............A.......G........................G.T............G.......T.G.......
```

```
20500B_2-4          .......--...........C....A.................G..A...G............A....T.........................G.......
20500B_1-3          .. ....--...........G.............A.......G........................A.A........A.............G.......
20500B_1-6          .......--...........G.............A.......G........................A.......A..............T.G.......
20500B_2-12         .......--...........G.............A.......G........................A........................G.......
20500B_2-9          .......--...........G....................AG........................A........................G.......
20500B_2-7          .......--...........G.............A.C.....A..T.......A.............A........................G.......
20500B_2-15         ......C--..........TC....................AA.............G.....A....T..................G.....G....GTG
20500B_2-18         .......--........A..C..G.........A........A........................T..A.....................T.......
20500B_2-6          .......--...........C................A....G........................T.......-....A...........A..A...A
20500B_2-16         .......--...........C.A...................G.......A.........T......T........................G.......
20500B_1-4          .......--......G....C.A...................A.G.....A.........A......A..G...................T.G....A..
20500B_1-7          .......--......G....C.A...................A.G.....A................TA..G.......C.............G.......
20500B_2-10         T.-....--...........C.....................A...G.G...G..............T...................C...CG.......
20500B_2-13         .......--......G....C.A...................G.G.....A................A..G..........G.........CG.......
20500B_2-2          ---....TG...........C........T............G........................T........................C.......
20500B_2-3          ....A..---------------A...................G........................TA.A....AAAA.-----.....A.G.......
CONSENSUS           GGgTGTG--GCTCgTaACACcTgATgACCAAACGgATCTACGgCaCCATCgAATCTCAAAcTtTAACtGccAAAAcTTtTGttTTTCATCctgTAGTCCc
                       * *                  * * * *    *  ***          *  *  *** *  **     **      *     *  **   ** *

                    201                                                                                              300
20500B_1-1          ..............................C--........CT............................CA...............G...........
20500B_1-2          ....T............A............C--........C..A...T......................C................GA..........
20500B_1-5          ...............T....T.........G--........C......T......................C................GA..........
20500B_2-19         ..............................T--...T....C.......T.....................C...A............A....A......
20500B_2-17         ..............................C--T.......T......T......................CA..............TG..TT.......
20500B_2-8          .............G................C--........T.....C..........T..A.........C.....G..........G...........
20500B_2-5          ........G.......-...T.........C--........C.........A....T..............T.....G.........AG...........
20500B_2-11         ................T.........C...T--.A......C.............................C.....G..........G........T..
20500B_2-14         ..............................T--..G.....C.......A.......A.............C................G...........
20500B_2-4          ...C..........................GGA........C......................TC.....C......T.........G....C......
20500B_1-3          ..........................GA..C--..G.....C.....G.......A...............C...G..T.........A...........
20500B_1-6          ..........................G...C--...C....C.......A.....A....A..........C...G..T.........A...........
20500B_2-12         ..............................T--........CT............A....A..........C...G..T.........A..........A
20500B_2-9          ...........................A..C--........CC.......A......A.............CA.......T......A...........
20500B_2-7          ..............................C--........C............T.............T..C................A...........
20500B_2-15         .....AT.......................C--........C.............................C................G....C......
20500B_2-18         ..............................C--......-.AT.................A..........T................G..G........
20500B_2-6          .....................C........G--....C...T......T......................C................G...T.C....A
20500B_2-16         .....A..G.......GTC.T....GG.T.-----......C..............T..............T...C..A.........A...........
20500B_1-4          ...............G...........T..GC-........C...T........A................C...............AG...........
```

```
205008_1-7        ..........G....G...........-..GC-........C..........................T......---------G..........
205008_2-10       ..............................C--.A......A.............G..AC..........C................G.......-..
205008_2-13       .................A............C--........A.......AT....C...C..........C..........T....G.....G..-..
205008_2-2        ...-.TT.........AA............C--........AT.........................T..G.........G...A....-------
205008_2-3        ...................T..........C--........CT.........................T................GA..........
CONSENSUS         TTTTAgAATTACTAAacgTCcTTTTTtgGGc--CgAGATTTccAGGAAcgCTTTCtCGGGgTTTGTGTGCAcgTAtTtcAAATCATAcggACCtaAAaAt
                  ***    * * ** *       ***     *        ***  *  *     **        **  ** **  *  *             *

                                                                                                   % identity with
                                                                                                    consensus
                  301                                                                        382
205008_1-1        ......T.......C.......C...........C....CA.........T.....C...C.................. 378  (96%)
205008_1-2        ......G.....T..C.......C...........C...--............G..C...C.................. 376  (96%)
205008_1-5        ...G..G.....T..C.......C...........C...--...A..........C...C.................. 376  (93%)
205008_2-19       ......T.......A.......C...........T....--A.............T...T............T...... 376  (94%)
205008_2-17       ......T.......A.......T......T....C...--...............C...C.................. 376  (94%)
205008_2-8        ......T.......C.......G...........C...--...............C...C.................. 378  (94%)
205008_2-5        ......T.......C...T...C...........C.A..--...............C...GA................. 375  (94%)
205008_2-11       ..A...T.......C.......A...........C...--...............C...C.................. 375  (95%)
205008_2-14       ......T.......C...  ...C...........C...--.........T....C...C.......A....T...... 375  (95%)
205008_2-4        .....ATT......C.......C....C......G...--...............C...C.C.............G.. 378  (94%)
205008_1-3        ......G.......C.......C...........T..TT--..........A...A...C.................. 375  (93%)
205008_1-6        ......G....A...C.......C...........T...T--A.............A...C.................. 376  (92%)
205008_2-12       ......G.......C.......C...........C...T--...............A...C.................. 375  (95%)
205008_2-9        ..--..T.......C.......C...........C...T--.........A.....CT..C.............T..... 374  (95%)
205008_2-7        ......T.......C.......C......A....C...--.........T....C...C.................. 375  (95%)
205008_2-15       ......T.....T..G.......C...........C...--...............C...C.................. 375  (93%)
205008_2-18       ......T.......A.......C...........C...--.........TA....C.A.C....T............. 375  (94%)
205008_2-6        ......T.......C.......A...........C...--.........T.....C...C.................. 374  (93%)
205008_2-16       ......T.......C.......C...........C...--...............C...CA.......C...T...... 372  (93%)
205008_1-4        ....T.T.......A.......CT.A........AA..T.--............T..C.A.T......T........... 377  (92%)
205008_1-7        ....T.T.......A.......C.......T..AA..T.--...............C.A.T......T........... 367  (92%)
205008_2-10       ......T.CA....A.......  ...........C...--....G.........----------........G..... 363  (92%)
205008_2-13       ......C.......A......T  ...........C...--....G.....T....----------....C...G..... 364  (92%)
205008_2-2        .G....T.......A.......C.A.........AC.A..--A..G...C.......C...CA......A........... 367  (90%)
205008_2-3        ......C.......C..C....C...........C...--.........T.....T...C.AT................ 359  (88%)
CONSENSUS         TAGAAGtCATTCcATcTTTATTCcCCTAGAGCCTTccAGcA--gTTCCTAAAccGCTCcagTcggaGcACGTTATcaTTCTT 376
                  *         *  ** **  **     * **  **  *      **  ** *    **          *    **  ** **
```
